# 'Genome order index' should not be used for defining compositional constraints in nucleotide sequences - a case study of the Z-curve

**DOI:** 10.1186/1745-6150-5-10

**Published:** 2010-02-17

**Authors:** Eran Elhaik, Dan Graur, Krešimir Josić

**Affiliations:** 1 McKusick - Nathans Institute of Genetic Medicine, Johns Hopkins University School of Medicine, Baltimore, MD 21205, USA; 2 Department of Biology & Biochemistry, University of Houston, Houston, TX 77204-5001, USA; 3 Department of Mathematics, University of Houston, Houston, TX 77204-3008, USA

## Abstract

**Background:**

The Z-curve is a three dimensional representation of DNA sequences proposed over a decade ago and has been extensively applied to sequence segmentation, horizontal gene transfer detection, and sequence analysis. Based on the Z-curve, a "genome order index," was proposed, which is defined as *S *= *a*^2^+ *c*^2^+*t*^2^+*g*^2^, where *a*, *c*, *t*, and *g *are the nucleotide frequencies of A, C, T, and G, respectively. This index was found to be smaller than 1/3 for almost all tested genomes, which was taken as support for the existence of a constraint on genome composition. A geometric explanation for this constraint has been suggested. Each genome was represented by a point *P *whose distance from the four faces of a regular tetrahedron was given by the frequencies *a*, *c*, *t*, and *g*. They claimed that an inscribed sphere of radius *r *= 1/ contains almost all points corresponding to various genomes, implying that *S *<*r*^2^. The distribution of the points *P *obtained by *S *was studied using the Z-curve.

**Results:**

In this work, we studied the basic properties of the Z-curve using the "genome order index" as a case study. We show that (1) the calculation of the radius of the inscribed sphere of a regular tetrahedron is incorrect, (2) the *S *index is narrowly distributed, (3) based on the second parity rule, the *S *index can be derived directly from the Shannon entropy and is, therefore, redundant, and (4) the Z-curve suffers from over dimensionality, and the dimension stands for GC content alone suffices to represent any given genome.

**Conclusion:**

The "genome order index" *S *does not represent a constraint on nucleotide composition. Moreover, *S *can be easily computed from the Gini-Simpson index and be directly derived from entropy and is redundant. Overall, the Z-curve and *S *are over-complicated measures to GC content and Shannon *H *index, respectively.

**Reviewers:**

This article was reviewed by Claus Wilke, Joel Bader, Marek Kimmel and Uladzislau Hryshkevich (nominated by Itai Yanai).

## Background

The nucleotide composition of genomes varies dramatically between and among taxa. The GC content is the primary measure to characterize genomic regions in terms of homogeneity, compositional bias, and compositional constraints [1].

Zhang and Zhang [2] proposed the Z-curve, an extension to the GC content measure, based on a three coordinate system of *x, y*, and *z*:(1)

where *a*, *c*, *t*, and *g *are the frequencies of the four nucleotides in a sequence. For instance, in the case of the sequence ACGTCGCG, the three coordinates are (0,0,-0.5).

Since it was first proposed, the Z-curve has been used in many applications of sequence segmentation [3-5], horizontal gene transfer detection [6], isochoric domain inference [3,5], and sequence analysis [7].

We study the characteristics of the Z-curve using a statistical quantity, the "genome order index" *S*, proposed to measure nucleotide composition. Zhang and Zhang [7] defined *S *as(4)

where *a*, *c*, *t*, and *g *are the frequencies of the four nucleotides in a sequence. It was observed [7,8] that *S *is negatively correlated with the Shannon entropy *H *[9], defined as(5)

In response to our comments [10], Zhang [11] argued that *S *is also "a linear transformation" of the Gini-Simpson index *GSI*, defined as(6)

Since *GSI *= *S *+ 1, for all intents and purposes, these two measures are the same. Zhang and Zhang [7] calculated *S *for 809 genomes of different species and found that *S *< 1/3 for all but two genomes. They claimed the limited observed range of *S*, from 1/4 to 1/3, supported the existence of a new constraint on nucleotide composition [8].

**Figure 1:**
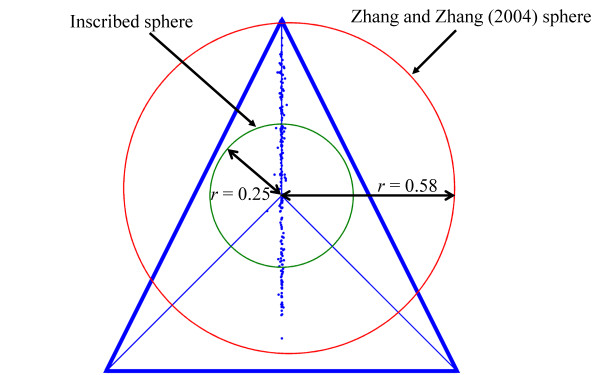
**Points of 235 bacterial genomes mapped to a regular tetrahedron with a height of 1 and edge lengths of /2**. Here, the face of the tetrahedron towards the observer is flat against the plane of projection. The points are mapped according to Z-curve coordinates with an origin in the center of the tetrahedron. The two spheres in the figures are an inscribed sphere of a regular tetrahedron with a radius of 0.25 and the sphere calculated by Zhang and Zhang [7]. Forty five percents of the points *P *are found outside the inscribed sphere thus violating the "constraint."

Zhang and Zhang [7] used a geometric argument to support this claim further. Each genome was mapped onto a point *P *inside a regular tetrahedron of height 1, and edge length /2 using simplicial coordinates. The four tetrahedral faces represent the four nucleotides, and the distances of *P *to the four faces equal the frequencies of A, C, T, and G, respectively. The sum of the distances from *P *to the four faces equals the height of the tetrahedron, a constant. For example, the sequence ACGTCGCG is mapped onto a point *P *with the simplicial coordinates (0.125, 0.375, 0.125, 0.375), representing the distances between *P *and the four tetrahedral faces. The position of each point *P *was calculated again in a Cartesian coordinate system with its origin at the center of the tetrahedron (see also [2]). Finally, *S *was calculated for all 809 points according to Eq. (4). For example, *S *= 0.3125 for the sequence ACGTCGCG. Zhang and Zhang [7] claimed that almost all points are located within a sphere inscribed in a regular tetrahedron so that *S *<*r*^2^. The radius *r *of the inscribed sphere in the case of a regular tetrahedron was calculated as 1/ (see Figure 1), so that *S *< 1/3. This inequality was presented as a new biological constraint.

The "genome order index" was selected as a case study to the usefulness of the Z-curve method. We show that the inscribed sphere calculations were erroneous and that the "conversion" from simplicial coordinates to Z-curve coordinates is misleading. Further, we show that *S *is a narrowly distributed measure of nucleotide composition and use the second parity rule to show that it can be derived directly from the Shannon entropy. Therefore, any constraints on *S *follow from constraints on *H*. Finally, we show that the Z-curve suffers from over dimensionality and that the only informative dimension is equivalent to the GC content measure.

## Results and Discussion

### "Inscribed sphere" or "circumscribed sphere"?

We first note that a regular tetrahedron of height 1 has an inscribed sphere of radius 0.25 rather than 1/ ≈ 0.58. The center of the tetrahedron is defined as the intersection of two space heights. A sphere of radius 1/ at the center nearly encompasses the tetrahedron (Figure 1). Hence, the conclusion that almost all genome mapping points *P *are located within an inscribed sphere of *r *= 1/ and thus follow *S *<*r*^2 ^is a consequence of a mathematical error.

In Figure 1, we present the *x *and *z *coordinates of 235 full bacterial genomes, which have very diverse GC content ranging from 0.22 to 0.77. We found that 45% of the bacterial genomes fall outside the actual inscribed sphere. Moreover, points *P *in the simplicial space are unrelated to the points calculated by the Z-curve. Since the only relevant coordinates are the Z-curve coordinates, which can be calculated directly from the data, the graphical representation of points *P*, using a tetrahedron is misleading and provides no additional information.

### *S *is narrowly distributed

**Figure 2:**
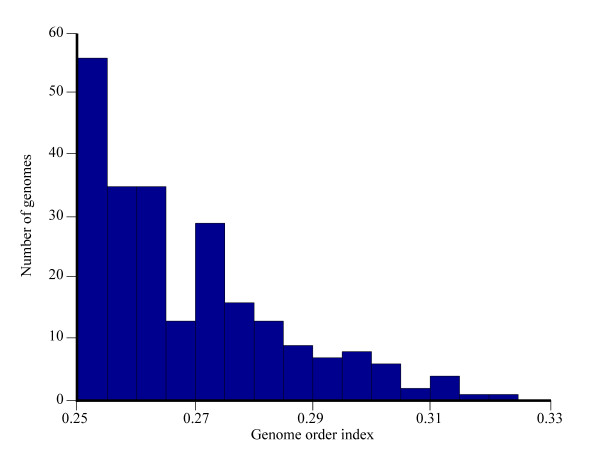
**Histogram of the genome order index *S *for 235 bacterial genomes**.

According to Zhang and Zhang [7], *S *< 1/3. This result was surprising because, in theory, the values of *S *can range between 0.25 and 1. We next show that the range of *S *is expected to be much narrower. We calculated *S *values for 235 bacterial genomes (Figure 2) and found that the distribution of *S *values follows an exponential distribution, as in Zhang and Zhang [7].

**Figure 3:**
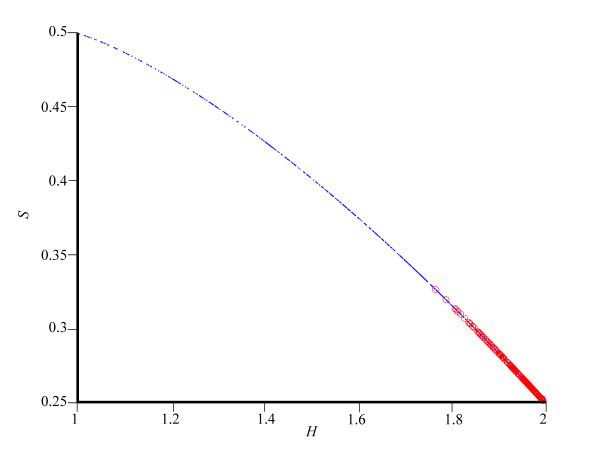
**Genome order index *S *versus *H *for 235 bacterial genomes (circles) and for 1,000 simulated genomes (dots)**. The bacterial genome at (1.76, 0.32) has the smallest GC content of 0.22.

Chargaff's second parity rule [12] states that for sufficiently large (>1,000 bp) single DNA strands, the number of A's approximately equals the number of T's, and the number of C's approximately equals the number of G's. Mitchell and Bridge [13] showed that the second parity rule holds for all double stranded DNA sequences in over three thousands viral, bacterial, archaeal, and eukaryotic genomes, with the exception of organelle genomes. When this rule is taken into account, we found that *S *can range only between 0.25 and 0.5 (see details in Appendix). Thus, since the second parity rule implies that certain nucleotide frequencies, such as those used by Zhang [11], are not attainable in practice, the potential range of *S *for real genomic sequences is much narrower than that proposed in Zhang and Zhang [7]. Figure 3 illustrates the narrow range of *S *versus *H *for 235 bacterial genomes and for simulated data. We note that the only detectable constraint is that determined by the empirical GC content of genomes, which in nature ranges approximately between 0.22 and 0.77. The GC content distribution delimits *S *to a range between 0.25 (GC content of 0.50) and 0.33 (GC content of 0.22). The second parity rule was similarly used by Zhang [8] to study the relations between *S *and *H*. In agreement with Mitchell and Bridge [13] we note that, although it is unknown which rule dictates the genome composition of single stranded genomes, it cannot be assumed that they are constrained by *S*.

### *S *equals to the Gini-Simpson index and is equivalent to *H*

It is easy to show that *S *is equal to *GSI *- 1 (Eq. 4 and 6), and is, thus, redundant. Subsequently, we show that under the assumption of the second parity rule, *S *can be derived directly from the Shannon entropy *H*, which we express as(7)

assuming that the frequencies of *a *and *t *are equal, as are *c *and *g*. Using Eq. (A1-A6), *S *is directly related to *H *according to(8)

where *S *ranges from 0 to 0.5. Zhang and Zhang [7] claimed that *S *"is a kind of negative *H *function," and that "*S *is negatively correlated with the Shannon *H *function [entropy], but with a simpler form and clear geometrical meanings." Eq. (8) shows that *S *is completely determined from the entropy and the two are correlated, contrary to Zhang [11], but in agreement with Zhang [8]. Moreover, *S *is less informative than *H *since it cannot be interpreted directly in an information theoretical or geometric sense, and it does not have the useful mathematical properties of *H*, such as additivity [11]. We note that the relation between *S *and *H *given in (Eq. 8) may not hold for DNA sequences that violate the second parity rule, such as organellar DNA and single stranded DNA sequences [13]. However, even these genomes obey a less stringent rule: that the number of *a*+*g*'s approximately equals the number of *t*+*c*'s, and therefore they cannot be used as evidence that *S *does not derive from *H*.

### The over dimensionality of the Z-curve

**Figure 4:**
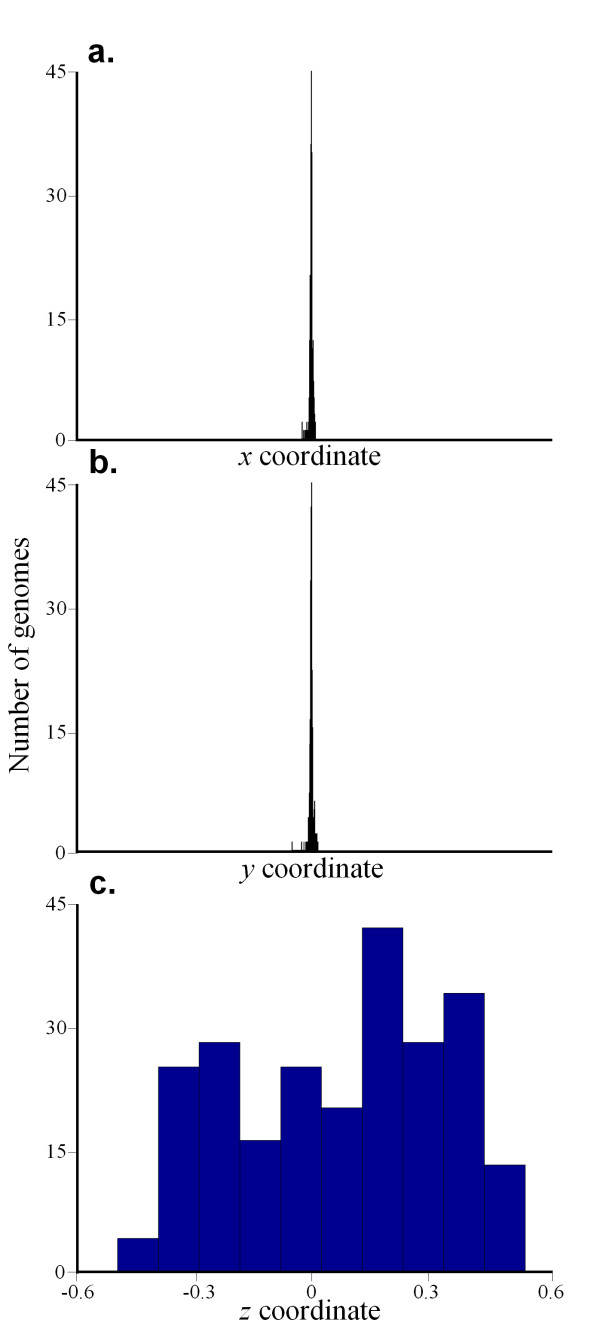
**Histograms of the three coordinates of the Z-curve for 235 bacterial genomes representing the difference between the nucleotides (a) AG and CT, (b) AC and GT, and (c) AT and GC**.

Zhang and Zhang [7] claimed that three dimensions are required to study the geometric properties of *S *using the Z-curve. Here we show that the Z-curve suffers from over dimensionality. In Figure 4, we show three histograms of the distributions of *x*, *y*, and *z *in 235 bacterial genomes. We used principal components analysis to calculate the coefficient of variation [14] and reduce the dimensionality of the dataset. The principal component variance of *z *is 0.07, compared to ~10^-5 ^for both *x *and *y*. Moreover, 99.91% of the variance is accounted for by the *z *coordinate and the *x *and *y *coordinates accounted for 0.053% and 0.003% of the variance, respectively, as expected from Chargaff's second parity rule [[12], see also Appendix]. The *z *axis is, therefore, the only meaningful coordinate for studying nucleotide composition. We note that the *z *axis is perfectly correlated with the GC content measure:(9)

The Z-curve, therefore, also appears to provide redundant information.

## Conclusions

The Z-curve, a three dimensional representation of DNA sequences, was proposed to characterize isochoric segments [3,15] and complete genomes [2,7]. Recently, Zhang and Zhang [7] suggested that a measure of DNA composition, the genome order index *S*, is a constraint for genome compositions. The authors used the Z-curve with a geometric explanation to support their arguments. The relation between *S *and the Shannon entropy *H *were further studied by Zhang [8].

By showing that the geometric representation was in error and that the range of values for *S *is narrower than originally claimed, we conclude that *S *does not represent a constraint on nucleotide composition. Moreover, we point at the limited usefulness of the Z-curve method to support the calculations of *S*. Next, we show not only that *S *can be easily computed from the Gini-Simpson index, but it can also be directly derived from entropy and is redundant. Finally, using principal component analysis, we show that only one out of three components of the Z-curve is important. Not surprisingly, the most useful component of the Z-curve is the GC content measure (*z*) that is already being used widely in genomic studies. The other dimensions of the Z-curve (*x *and *y*) contribute less than 1% of the variance and would be of little use in most studies but can be employed to study deviations from the second parity rule. Overall, we must conclude that both the Z-curve and *S *are over-complicated measures to GC content and Shannon *H *index, respectively.

## Methods

We used two datasets of nucleotide frequencies based on real and surrogate data. For the first dataset, we used 235 bacterial genomes that were downloaded from NCBI website http://www.ncbi.nlm.nih.gov/. The list of species can be found in Table S1 (Additional file 1). For the second dataset, we generated 1,000 genomic sequences with nucleotide frequencies drawn from a uniform distribution ranging from 0 to 0.5 using Eq. A1 and A2.

## Competing interests

The authors declare that they have no competing interests.

## Authors' contributions

EE performed the analyses and wrote the draft manuscript. KJ and DG contributed to the interpretation of the results and the final version.

## Appendix

### Detailed steps showing that *S *is narrowly distributed

According to Chargaff's second parity rule(A1)

Following this rule, it is possible to write the sum of *a *and *c *frequencies as(A2)

and both *a *and *c *range from 0 to 0.5. Eq. (4) can, therefore, be rewritten as(A3)

where *S *ranges from 0.25 to 0.5. Eq. A3 has two solutions for *a*(A4)

Shannon's *H *can also be rewritten as(A5)

where *H *ranges from 1 to 2. Solving Eq. A5 with Eq. 4 solution gives an expression for *H *that depends on only on *S*(A6)

where .

## Reviewers' comments

### Reviewer's report 1

Review by Claus Wilke, Institute for Cell and Molecular Biology, University of Texas

This is a brief theoretical work that addresses whether a barycentric coordinate system can provide a useful characterization of the four nucleotide frequencies in a DNA sequence. Specifically, the authors investigate whether such a coordinate system can provide insight into constraints on nucleotide frequencies beyond just GC content. The authors find that this is not the case, contrary to claims made in the literature.

The article is clearly written and convincing. I agree with all claims made in this article.

### Reviewer's report 2

Review by Uladzislau Hryshkevich and Itai Yanai, Department of Biology, Technion - Israel Institute of Technology

Elhaik et al. and Zhang & Zhang are involved in a dispute stemming from a publication by the latter group in 2004. Given the situation as it is presented by Elhaik *et al. *we find that the described results support the conclusions. Most convincingly for us is the lack of information derived from the x- and y- axes of the Z-curve. Given this lack of x and y variation for known genomes, we agree with Elhaik *et al. *that the Z-curve contains excessive dimensions. The z-axis is the only one that contains variation which Elhaik *et al. *show is reflective of the GC-content. Thus, while the Z-curve is a mathematically elegant approach to reveal compositional constraints, real genomes apparently do not scatter within this defined space sufficiently well to warrant its use. Rather, genome DNA composition follows Chargaff's 2nd parity rule which consequently makes the GC-content the simplest indicator of compositional variation.

### Reviewer's report 3

Review by Joel Bader, Department of Biomedical Engineering, Johns Hopkins University

This manuscript describes methods to analyze nucleotide content and appears technically correct. It reconciles results that have been reported by other groups by providing a more thorough treatment.

### Reviewer's report 4

Review by Marek Kimmel, Department of Statistics, Rice University

The paper makes a conclusive point concerning inaccuracies in the definition and evaluation of properties of the so-called Z-curve by its authors and other sources uncritically citing them. All mathematical point, which are made, seem to be correct, with a particular emphasis concerning geometric arguments about spheres inscribed in a regular tetrahedron and over-dimensionality of the Z-curve.

There seems to be one intriguing point: The analysis of 235 bacterial genomes hints at the distribution of *S *values following exponential distribution. However, inspection of Figure 2 suggests that the distribution is imperfect, and it has a "dip" around the value *S *= 0.27. This might be an artifact of sampling. If so, then taking more genomes might fix it. On the other hand, if this is such simple distribution, should it be not computable from "first-principles" concerning for example the distributions of the GC/AT composition of the genomes?

#### Author's response

According to Chargaff's second parity rule, we can derive (see Eq. A3)

This quadratic function achieves a minimum exactly at *a *= 1/4, at which point *S *= 1/4. Therefore the apparent exponential distribution of *S *results from the observation that between genomes the distribution of the frequency of *a *has mean 1/4, and falls off away from this mean. Under the quadratic transformation that defines *S*, this distribution appears exponential: it is vanishes to the left of *S = 1/4*, and its fall-off to the right of *S = 1/4 *is accentuated. A distribution could be computed from a fitted distribution of nucleotide frequencies - something we do not attempt. Fluctuations due to limited sample size seem to be responsible for a dip in the distribution of frequencies of *a *and *t *around 0.32 and the frequencies of *c *and *g *around 0.18 (giving *S *= 0.27). This translates into a "dip" and adjacent "hill" observed in Figure 2. We are not aware of any such special properties of the actual nucleotide frequency distribution, so the trend observed in Figure 2 is likely due to sample bias.

## Supplementary Material

Additional file 1 **Table S1**. Size and GC content for 235 bacteria. Click here for file
